# *In vivo* adeno-associated virus-mediated LDLR/PCSK9 intervention for familial hypercholesterolemia

**DOI:** 10.1016/j.gendis.2025.101632

**Published:** 2025-04-10

**Authors:** Zeyu Han, Cheng Tan, Jianzhong Ai, Ye Zhu

**Affiliations:** a Department of Urology, Institute of Urology, West China Hospital, Sichuan University, Chengdu, Sichuan 610041, China; b Department of Cardiology, Institute of Cardiology, West China Hospital, Sichuan University, Chengdu, Sichuan 610041, China

Familial hypercholesterolemia is an autosomal dominant disorder characterized by a significant elevation of total cholesterol, especially low-density lipoprotein cholesterol (LDL-C).^1^ Mutations in the function of both LDL receptor (LDLR) and proprotein convertase subtilisin kexin 9 (PCSK9) genes are responsible for familial hypercholesterolemia.^2^ It is not yet clear whether the simultaneous intervention of both genes can have a synergistic effect on achieving further lipid-lowering goals. We investigated whether dual-target intervention could further reduce lipid levels and achieve improved therapeutic outcomes using adeno-associated virus (AAV) to overexpress the LDLR gene and by simultaneously performing knocking of the PCSK9 gene.

The overexpression vector was created through homologous recombination cloning of the mouse LDLR (NM_010700) transcript, amplified by PCR, and the linearized plasmid AAV-multiple cloning site vector digested by enzymes (Fig. S1A). One-generation sequencing was conducted to validate the overexpression sequence post-vector construction. The success of vector construction was confirmed through a four-stage PCR. For PCSK9 genome editing, three target guide RNA (gRNA) sequences were designed, targeting PCSK9 transcripts 1, 5, and 3, respectively (Fig. S1B). After enzymatic cloning, one-generation sequencing was performed for the gRNA sequences, and sequence comparison confirmed the successful construction of knockout vectors (Fig. S1C). The pAAV-EF1a-enhanced green fluorescent protein (eGFP) fluorescent control was used to transfect cells to verify the transfection effect. Nano-liposome reagent was applied for transfection of Hepa1-6, H2.35, and Neu-2a cell lines, resulting in fluorescence area and intensity consistent with the study at 48 h (Fig. S2). Subsequent transfection of PX601 plasmid and GFP control resulted in significant SaCas9 protein expression in Hepa1-6 cells after 48 h (Fig. 1A). Transfecting H2.35 cells with the three PCSK9 knockout vectors (Z1, Z2, Z3) and the fluorescent control. Lanes 1, 2, and 3 displayed weaker post-gene editing enzymatic bands compared with control lane 4 (Fig. S3A). Clear gene editing enzyme cut bands were observed in cells transfected with the Z2 and Z3 vectors (Fig. S3B), indicating efficient gene editing in the Neu-2a cell line. Proteins extracted from Neu-2a cells post-transfection with the PCSK9 knockdown vector showed a significant decline in PCSK9 expression after Z2 vector transfection (Fig. 1B). The Z2 vector was selected for subsequent AAV virus packaging. The pAAV-CMV-mLDLR was transfected into 293T cells, with a 48-h cell collection revealing significant mLDLR expression through quantitative PCR and western blotting (Fig. 1C, D).

**Figure 1:**
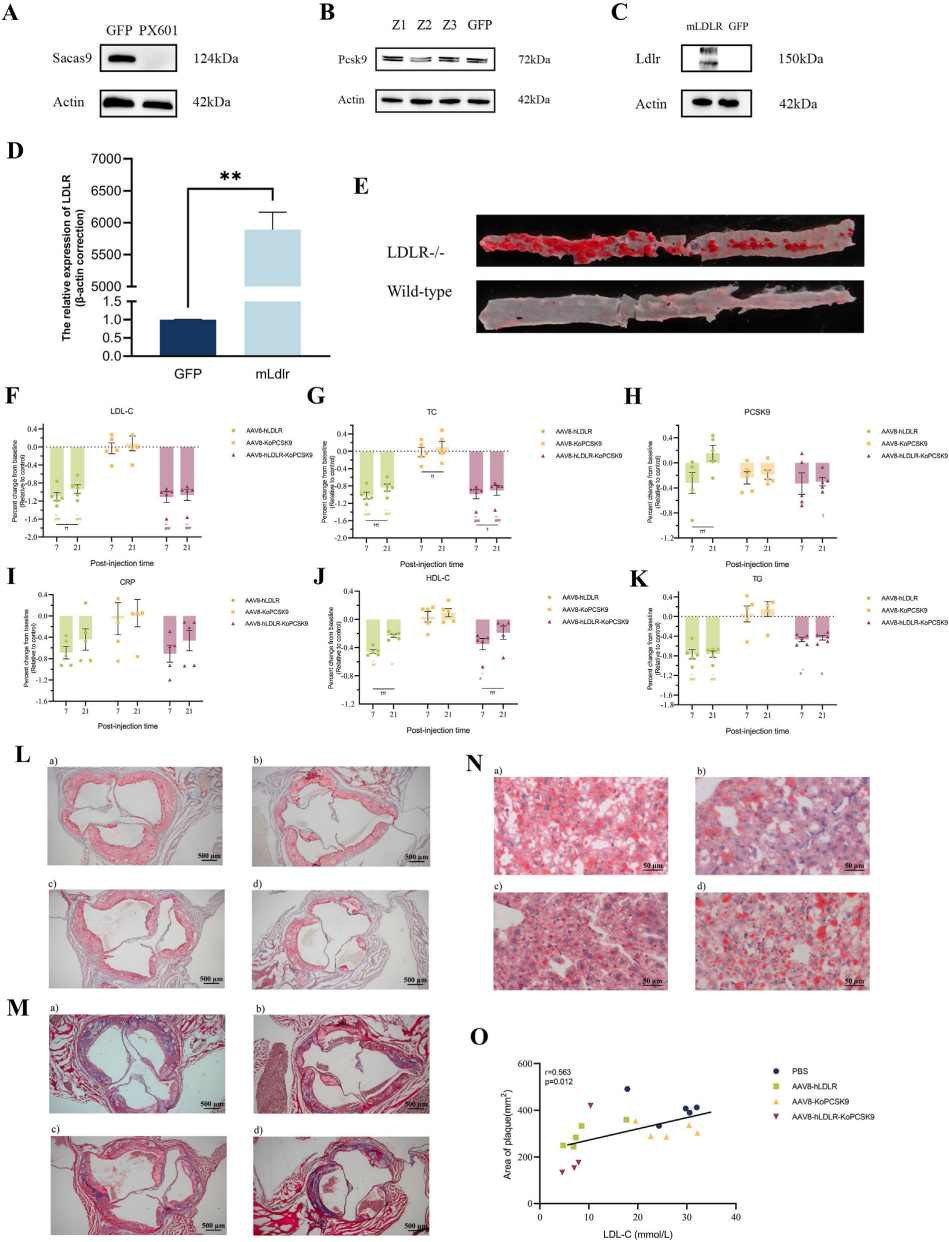
*In vivo* adeno-associated virus (AAV)-mediated LDLR/PCSK9 intervention for familial hypercholesterolemia. **(A)** Detection of *Staphylococcus aureus* Cas9 (SaCas9) protein expression 48 h after transfection of Hepa1-6 cells with the PX601 plasmid, as assessed by western blotting. **(B)** Western blotting analysis of PCSK9 protein expression in Neu-2a cells after transfection with the PCSK9 knockdown vector (Z2) showed a significant reduction in protein levels compared with the control. **(C)** Western blotting results demonstrated successful expression of the mouse LDLR (mLDLR) protein in 293T cells after transfection with the pAAV-CMV-mLDLR vector, confirming the overexpression of the receptor. **(D)** PCR assay of 293T cells following transfection with pAAV-CMV-mLDLR, confirming the amplification of mLDLR transcript. **(E)** Macroscopic oil red O staining of dissected aortas from LDLR^−/−^ mice on a high-fat diet. Significant plaque accumulation is evident in the aortic walls of LDLR^−/−^ mice, indicating advanced atherosclerosis compared with wild-type C57 mice on a normal diet. **(F)** Percent change of low-density lipoprotein cholesterol (LDL-C) levels relative to baseline. The AAV8-hLDLR group showed a significant reduction in LDL-C levels at both 7 and 21 days post-treatment, with a notable treatment-by-time interaction. The AAV8-hLDLR and AAV8-hLDLR-KoPCSK9 groups exhibited the greatest decrease in LDL-C levels, significantly different from the PBS and AAV8-KoPCSK9 groups. **(G)** Percent change of cholesterol (CHO) levels relative to baseline. Both the AAV8-hLDLR and AAV8-hLDLR-KoPCSK9 groups showed significant reductions in CHO levels at 7 and 21 days, with a clear treatment-by-time interaction. The AAV8-KoPCSK9 group did not show significant changes in CHO levels compared with the PBS control, indicating that PCSK9 knockdown alone did not significantly affect CHO levels. **(H)** Percent change of PCSK9 levels relative to baseline. Serum PCSK9 levels in mice treated with AAV8-hLDLR, AAV8-KoPCSK9, AAV8-hLDLR-KoPCSK9, or PBS control. **(I)** Percent change of C-reactive protein (CRP) levels relative to baseline. CRP levels at baseline and 7 days across the treatment groups. **(J)** Percent change of high-density lipoprotein cholesterol (HDL-C) levels relative to baseline. HDL-C levels at baseline and 7 days in all treatment groups. **(K)** Percent change of triglyceride (TG) levels relative to baseline. TG levels at baseline, 7 days, and 21 days across groups. **(L)** Oil red O staining of aortic root plaque. Lipid accumulation in aortic root plaques across all treatment groups. **(M)** Masson staining of aortic root plaque. Fibrosis in aortic plaques across treatment groups. **(N)** Oil red O staining of the liver. Hepatic lipid content across all treatment groups. Red lipid droplets were observed between the four groups, representing lipid accumulation in regional hepatocytes. **(O)** Correlation between LDL-C levels and plaque area. Correlation between serum LDL-C and aortic plaque area across groups. The control group was the correction group. a, PBS group; b, AAV8-hLDLR; c, AAV8-KoPCSK9; d, AAV8-hLDLR-KoPCSK9; ∗, compared with the PBS control group; #, compared with the KoPCSK9 group; ‡, compared with the hLDLR group; †, 7-day versus 21-day percent change. The error line indicates the standard error of the mean. ∗*p* < 0.05, ∗∗*p* < 0.01, ∗∗∗*p* < 0.001.

A high-fat diet was given to the LDLR^−/−^ mice starting in week 10, and at week 16, the atherosclerosis model was evaluated. Compared with C57 mice on a normal diet, high-fat-fed LDLR^−/−^ mice displayed significant alterations in lipid profiles, featuring notably elevated levels of LDL-C, cholesterol, high-density lipoprotein cholesterol (HDL-C), and triglyceride (Figs. S4A–D). Macroscopic oil red O staining of dissected mouse aortas revealed substantial red regions in the adipose tissue of LDLR^−/−^ mice, indicating significant lipid accumulation in these areas (Fig. 1E). In LDLR^−/−^ mice, the absence of LDLR prevents the normal clearance of LDL from the bloodstream, resulting in disrupted cholesterol metabolism. Twenty LDLR^−/−^ mice were randomly grouped, and after establishing baseline data, phosphate-buffered saline (PBS), AAV8-hLDLR, AAV8-KoPCSK9, and AAV8-hLDLR-KoPCSK9 treatments were administered. Serum assays were collected at 7 days and 21 days, with LDL-C and cholesterol as primary treatment targets. Both the hLDLR and hLDLR-KoPCSK9 groups exhibited significant reductions in LDL-C and cholesterol levels at either 7 or 21 days, significantly differing from the PBS control and KoPCSK9 groups (Figs. S5A and B). PCSK9 knockdown alone did not affect the primary therapeutic targets and showed no difference from the PBS control group. Notably, a treatment-by-time interaction was observed for hLDLR treatment at the LDL-C level (Fig. 1F). While at the cholesterol level, hLDLR, KoPCSK9, and hLDLR-KoPCSK9 showed a treatment-by-time interaction (Fig. 1G). Considering PCSK9 as one of the therapeutic targets, serum PCSK9 levels did not exhibit significant changes (Fig. 1H; Fig. S5C). Additionally, we assessed the C-reactive protein, detecting treatment-related differences only at 7 days (Fig. 1I; Fig. S5D). Although HDL-C and triglyceride were not the main intervention indicators, treatment effects on HDL-C and triglyceride were as anticipated. At 7 days, the percentage change in HDL-C in the hLDLR and hLDLR-KoPCSK9 groups significantly differed from the PBS control and KoPCSK9 groups (Fig. S5E). Simultaneously, there was a treatment-by-time interaction for both interventions in HDL-C levels (Fig. 1J). At the triglyceride level, disparities were observed between the hLDLR group and the hLDLR-KoPCSK9 group compared with both the PBS control group and the KoPCSK9 group at both 7 and 21 days (Fig. S5F). Notably, there appeared to be no treatment-by-time interaction (Fig. 1K). Liver function, evaluated by alanine aminotransferase, aspartate aminotransferase, and albumin, showed no differences in levels and percentage change relative to baseline between the groups before and after viral injection. Furthermore, no treatment-by-time interaction was observed in liver function, except for the hLDLR group, which exhibited a difference in the percentage change in ALT levels (Figs. S6A–C).

We conducted oil red O staining and Masson staining of aortic plaques to evaluate the therapeutic intervention on aortic root plaques. The accumulation of plaques in different groups as well as the fibrosis status within were illustrated in Figure L, M. Quantifying the plaques revealed differences between hLDLR-KoPCSK9 and PBS controls (Fig. S6D). Subsequently, we assessed the therapeutic effect on liver fat content using oil red O staining (Fig. 1N). No differences in liver fat content were observed between treatment groups, both in terms of the oil red O area and the percentage of oil red staining per unit of liver (Figs. S6E and F). The correlation between lipid levels and plaque area was explored. Both LDL-C and triglyceride levels exhibited a linear regression relationship with plaque area, with a stronger association observed between LDL-C and plaque area (*r* = 0.563) (Fig. 1O; Fig. S5G). However, no correlation was found between serum PCSK9 levels, HDL-C levels, and plaque area (Figs. S6H and I).

The study observed significant changes in the lipid profile of LDLR^−/−^ mice using AAV for targeted intervention in LDLR and PCSK9. However, we did not find a synergistic effect of simultaneous LDLR and PCSK9 intervention on lipid changes. Interestingly, on day 21 of monitoring the LDLR overexpression group, a significant rebound in serum PCSK9 protein levels was detected. This rebound phenomenon may be attributed to the massive clearance of lipids from the serum after LDLR function is restored, activating cholesterol regulatory element binding protein (SREBP2) and enhancing PCSK9 gene expression in the liver.^3^ The overexpression of LDLR and knockdown of PCSK9 effectively inhibited this rebound phenomenon. The lipid-lowering effects were not significantly observed with PCSK9 intervention alone in this study. This lack of efficacy may be attributed to the inefficiency of PCSK9 knockdown in our research. PCSK9 binds to the epidermal growth factor precursor of LDL-R, facilitating LDL-R degradation, with increased affinity under acidic conditions.^4^ While statins contribute to lipid reduction by enhancing LDL-R expression on hepatocyte surfaces, their negative feedback regulation can lead to an up-regulation of PCSK9 expression, potentially impeding the lipid-lowering effect.^5^

In conclusion, our study demonstrated that the overexpression of LDLR exhibited significant improvement in LDL-C, total cholesterol, and triglyceride levels in LDLR null mice. In the aortic valve plaque treatment assessment, dual gene intervention demonstrated favorable plaque improvement potential.

## CRediT authorship contribution statement

**Zeyu Han:** Data curation, Formal analysis, Investigation, Methodology, Software, Visualization. **Cheng Tan:** Data curation, Formal analysis, Software, Supervision, Validation. **Jianzhong Ai:** Conceptualization, Project administration, Supervision, Writing – original draft, Writing – review & editing. **Ye Zhu:** Conceptualization, Funding acquisition, Project administration, Supervision, Writing – original draft, Writing – review & editing.

## Ethics declaration

The animal study was reviewed and approved by the Animal Ethics Review Committees of the West China Hospital.

## Data availability

The authors of this article will make available the raw data that supports the conclusions presented herein.

## Funding

This study was supported by grants from the 10.13039/501100004829Science & Technology Department of Sichuan Province, China (No. 2020YFS0207 to Y.Z.).

## Conflict of interests

The authors declared that the research was conducted in the absence of any commercial or financial relationships that could be construed as a potential conflict of interest.
