# Supplementary material for: In vivo adeno-associated virus-mediated LDLR/PCSK9 intervention for familial hypercholesterolemia
Source: Genes Dis. 2025 Apr 10;12(6):101632. doi: 10.1016/j.gendis.2025.101632 (PMC12281168; doi:10.1016/j.gendis.2025.101632)
Supplement: Multimedia component 1 [file mmc1.docx]

**Title: *In vivo* adeno-associated virus-mediated LDLR/ PCSK9 intervention for familial hypercholesterolemia: a rapid communication**

**Author:** Zeyu Han^1#^, Cheng Tan^2#^, Jianzhong Ai^1*^, Ye Zhu^2*^

**Affiliation:** ^1^Department of Urology/Institute of Urology, West China Hospital, Sichuan University, 88 South Keyuan Road, Chengdu, 610041, China.

^2^Department of Cardiology, Institute of Cardiology, West China Hospital, Sichuan University, 88 South Keyuan Road, Chengdu, 610041, China.

^#^These authors contributed equally as the first author.

***Correspondence to**: Jianzhong Ai at Department of Urology, Institute of Urology, West China Hospital, Sichuan University, 88 South Keyuan Road, Chengdu, 610041, P. R. China. E-mail address: jianzhong.ai@scu.edu.cn or Zhu Y at Department of Cardiology, Institute of Cardiology, West China Hospital, Sichuan University, 88 South Keyuan Road, Chengdu, 610041, China. E-mail address: zhuye1974@163.com (Y. Zhu).

**Supplementary methods/materials:**

**This supplementary file shows the methods applied in supporting the results in the main text.**

**Cell culture, mice, and assays of LDL-C, HDL-C, TC, TG, ALT, AST, and albumin**

Mouse hepatocarcinoma cells Hepa1-6, mouse hepatocytes H2.35, mouse brain neuroma cells Neu-2a, and human embryonic kidney cells 293T were cultured at 37°C in a 5% CO2 humidified atmosphere. A complete medium containing 10% fetal bovine serum was utilized for cell resuscitation, passage, and other procedures. Cell transfection was executed using XfectTM Transfection Reagent. LDLR^-/-^ 8-week-old male mice, procured from the company, were initially provided with a normal diet. At week 10, a high-fat diet (40 kcal%, high cholesterol 1.25%) was introduced. Diet, water, and bedding were weekly changed, and body weight measurements were recorded throughout the high-fat feeding period. The atherosclerosis model was confirmed at 16 weeks of high-fat diet, and intervention injections were administered at 20 weeks of high-fat diet. Baseline data were collected before the intervention injection. For biochemical detection, mouse serum was obtained using the tail-tip blood sampling method. A 200 μL blood sample was collected, allowed to stand for 30 minutes at room temperature, and subsequently centrifuged at 3000 rpm. The supernatant was stored at 0°C. Subsequently, 50 μL of serum was diluted five times in a PBS solution for biochemical analysis. The levels of LDL-C, HDL-C, TC, TG, ALT, AST, and albumin were assayed to evaluate the biochemical profile.

**Construction of AAV-CMV-mLDLR overexpression vector**

To achieve mouse LDLR gene overexpression, the NM_010700 transcript with the maximum base count was selected, and the pAAV-MCS vector served as the platform. Downstream restriction sites of the CMV promoter in the ITR expression frame were scrutinized for vector linearization, with BamHI and HindIII selected as the restriction sites. The overexpression vector was synthesized through homologous recombination. The vector backbone double digestion system included 1 µg pAAV-MCS plasmid, 5 µL rCutSmarTM Buffer, 1 µL BamHI HF endonuclease, 1 μL HindIII HF endonuclease, and ddH2O to a total volume of 50 μL. Enzyme digestion occurred by incubating for one hour at 37°C. Gel recovery of the linearized carrier was conducted using the MiniBEST Agarose Gel DNA Extraction Kit. We used SnapGene purpose gene PCR primer design:

Forward (F):

5'-GATTGAATTCCCCGGGGATCCGCCACCATGAGCACCGCGGATCTG-3'; Reverse (R):

5'-AGCGCTGCTCGAGGCAAGCTTTCATGCCACATCGTCCTCCAG-3'.

PCR amplification of the target gene utilized PrimeSTAR Max DNA Polymerase, with cDNA obtained from the murine H235 cell line serving as the amplification template. The PCR products underwent agarose gel electrophoresis on a 1% agarose gel, and gel recovery was performed to obtain the desired bands. The target gene was cloned into the linear vector through homologous recombination using the In-Fusion® Snap Assembly kit. After competent transformation, the overexpression vector was obtained through culture and screening. Plasmid extraction and sequencing were conducted using the MiniBEST Plasmid Purification Kit.

**AAV-CMV-SaCas9-U6-gRNA knockout vector construction**

The PX601 plasmid served as the foundational backbone for the construction of the knockout vector. Drawing on prior literature and the BROAD CRISPick high-throughput screening, three gRNA sequences were selected for the creation of PCSK9 knockout vectors:

Z1gRNA: 5'-CACCGCAGCCACGCAGAGCA-3'

Z2gRNA: 5'-CCGCTGACCACACACCTGCCAG-3'

Z3gRNA: 5'-AGCATCCCATGGAACCTGGA-3'

Each gRNA oligonucleotide carried the sticky end of BsaI was synthesized by the company, and diluted with TE buffer to achieve a concentration of 100 μM. The oligonucleotide sequences were:

Z1 strand F: 5'-CACCGCACCGCAGCCACGCAGAGCA-3'

Z1 strand R: 5'-AAACTGCTCTGCGTGGCTGCGGTGC-3'

Z2 strand F: 5'-CACCGCCGCTGACCACACACCTGCCAG-3'

Z2 strand R: 5'-AAACCTGGCAGGTGTGGTCAGCGGC-3'

Z3 chain F: 5'-CACCGAGCATCCCATGGAACCTGGA-3'

Z3 chain R: 5'-AAACTCCAGGTTCCATGGGGATGCTC-3'

The annealing process involved combining 1 μL of oligonucleotide chain F, 1 μL of oligonucleotide chain R, and 8 μL of TE buffer. The oligonucleotides underwent denaturation at 95°C using a PCR instrument, followed by a gradual temperature reduction for re-annealing. A 1 μL aliquot of the annealed oligonucleotide solution was diluted with TE buffer to create a 100 nM (100 fmol/μL) dilution. The PX601 plasmid was subjected to digestion, and the resultant product was recovered through gel extraction. Oligonucleotide dimers and the digested PX601 vector were ligated using the DNA Ligation kit. Subsequent steps included competent transformation and plasmid mini-extraction, following the methodology employed in overexpression vector construction

**RNA extraction, reverse transcription PCR, and Real-Time PCR**

RNA extraction was carried out utilizing the Total RNA Isolation Kit, and subsequent cDNA synthesis was performed through reverse transcription using RT EasyTM II. The reverse transcription system comprised 2 × RT OR-EasyTM Mix, 2 μL of RNA, and 3 μL of RNase-Free ddH_2_O. For fluorescence PCR quantification, cDNA was subjected to SYBR Green qPCR Mix. Primers for fluorescence quantification were sourced from Primer Bank. The fluorescence quantitative PCR configuration involved SYBR Green qPCR Mix (10 μL), 0.5 μL each of forward and reverse primers, 2 μL of cDNA template, and 7 μL of RNase-Free ddH2O.

**Western blotting**

Lysate, supplemented with preconfigured 100mM phenylmethanesulfonyl fluoride (PMSF) to achieve a final PMSF concentration of 1mM, was used for protein extraction. Each sample received 200 μL of lysate, underwent vortexing and centrifugation, and the supernatant was collected. Protein concentration was assessed via the BCA Protein Assay. MOPS-SDS Running Buffer, prepared using 1 L of deionized water, was used as the electrophoresis liquid. The electrophoresis process employed FuturePAGETM 4-20% precast gel and a polyvinylidene difluoride (PVDF) membrane. Skim milk powder, dissolved in TBST, was employed to create a 5% blocking solution. After the completion of membrane transfer, the PVDF membrane was immersed in the blocking solution for a 2-hour blocking period. The primary antibody was incubated at 4°C for an entire night after being diluted in blocking solution. Following primary antibody incubation, the bands underwent three washes, each lasting 10 minutes, using TBST. A secondary antibody, diluted in a 1:8,000 TBST solution, was applied for a 1-hour incubation. After a final set of three washes, each lasting 10 minutes, the ECL luminescent solution was prepared, and bands were exposed using a chemiluminescence instrument.

**Virus packaging and harvesting**

Plasmid extraction was conducted using the Plasmid Bulk Extraction Kit according to the manufacturer's protocols. To ensure optimal density during transfection, 293T cells were inoculated in T225 culture flasks one day before the procedure. On the day of transfection, Xfect Transfection Reagent was employed to cotransfect three plasmids: 53 μg of packaging plasmid, 53 μg of Rep Cap 2/8, and 53 μg of helper plasmid, supplemented with Xfect Reaction Buffer. After six hours, 40 ml of fresh complete medium replaced the initial medium. Viruses were harvested 56 h after transfection. For virus collection, 500 μL of 0.5 M EDTA (pH 8.0) was added to a T225 culture flask, allowing it to stand for 10 minutes at room temperature. Cells were gathered into a 50 ml sterile centrifuge tube, and the supernatant was removed by centrifuging the tube for 10 minutes at 1700 rpm. AAV Extraction Solution facilitated the extraction of the virus. The cells in the tube were vortexed to loosen them, and 2 ml of AAV Extraction Solution A was added. After vortexing and standing for 3 minutes, the process was repeated three times. The supernatant was aspirated to a new sterile centrifuge tube, and 200 μL of AAV Extraction Solution B was added. Vortexing and immediate centrifugation followed, turning the supernatant pink.

**AAV titration determination**

AAV was purified and concentrated using CsCl. AAV virus titer was determined using AAVpro® Titration. The decontamination system involved 2 μL of AAV virus liquid, 2 μL of 10X DNase I Buffer, 1 μL of DNase I, and 15 μL of ddH_2_O. DNase I was inactivated by heat treatment at 95°C for 10 minutes. Inactivation involved the addition of 20 μL of Lysis Buffer at 70°C for 10 minutes. AAV genome was then diluted using 40 μL EASY Dilution for fluorescence quantitative PCR assay. A standard curve was created with a gradient dilution of the positive control (2 × 10^7^ copies/μL) to concentrations of 2×10^7^, 2×106, 2×10^5^, 2×10^4^, 2×10^3^, and 2×10^2^. A 50X Primer mix was configured with AAV Forward Titer Primer (5 μL), AAV Reverse Titer Primer (5 μL), and ddH2O (15 μL). The PCR reaction solution was prepared with TB Green PremixExTaq II (12.5 μL), 50X Primer mix (0.5 μL), ddH_2_O (7 μL), and template (5 μL). PCR reaction, standard curve plotting, and viral titer calculation were then performed.

**Enzyme-linked Immunosorbent Assay (ELISA)**

The Mouse PCSK9 ELISA kit was utilized for the quantification of PCSK9 protein concentration in serum. Double-distilled water was used to dilute the concentrated wash solution at a 1:24 ratio. Following a 10-minute centrifugation at 10,000 g for one minute, the lyophilized standard was vortexed, dissolved, and mixed with one milliliter of sample diluent. A standard working solution was prepared with a final concentration of 4000 pg/mL following transient centrifugation. Each concentration was obtained by multiple dilutions, with 0 pg/mL used as the blank control. For testing, 2 μL of diluted serum was used, diluted 1,000 times with sample diluent. Then, 100 μL of standard or the test sample was added, mixed with light shaking, and incubated at 37°C for 90 minutes. Biotin antibody working liquid (100 μL per well) was added and incubated at 37°C for 1 hour. After that, 100 μL of HRP enzyme combination working liquid was added to each well, and the mixture was incubated for 30 minutes at 37°C. After adding 90 μL of substrate TMB solution, the incubation continued at 37°C in the dark for 20 min. Following the termination of the reaction, the absorbance value was measured using a microplate reader. The blank control was used to correct the absorbance value, and a standard curve was created to determine the PCSK9 content in the samples.

**Oil red O staining**

Staining was conducted using a modified Oil Red O staining kit. Before staining, Oil Red O staining solution A and solution B were mixed in a 3:2 ratio to prepare the staining solution, which was used on the spot after standing for 10 minutes. Frozen sections or dissected aorta were washed in distilled water. After immersion in 60% isopropanol for 30 seconds, the samples were placed into the prepared staining solution and stained for 15 minutes under closed conditions. Dyed samples were washed to remove the dye solution in 60% isopropyl alcohol, followed by distilled water to clean. A hematoxylin staining solution was used for 2 minutes (for frozen sections) and washed with distilled water. After blotting moisture, glycerin gelatin was used for sealing.

**Masson staining**

An improved Masson trichromatic staining kit was used for staining. Frozen pieces were dyed with a mordant and then mordanted for an hour at 60°C. They were then rinsed in distilled water for ten minutes. After staining for 3 minutes, the sections were submerged in a lapis lazuli blue staining solution and twice rinsed with water for 15 seconds each time. The sections were stained for three minutes with hematoxylin staining solution, and then they were twice rinsed with water for 15 seconds each time. Following 10 seconds of differentiation, the cells were rinsed with distilled water for 10 minutes. The slides were then dripped with Ponceau stain solution and washed with water for 15 seconds after 10 minutes of staining, twice with water. The phosphomolybdic acid solution was added drop by drop and incubated for 10 minutes. Following liquid removal, aniline blue staining was applied for 5 minutes. The excess aniline blue solution was washed off with a weak acid solution, and the sections were covered by dropping the weak acid working solution, with the reaction lasting for 2 minutes. After 30 seconds of dehydration with 95% ethanol, dehydration was performed twice using absolute ethanol for 30 seconds and 1 minute. Xylene was employed for transparency twice, with each instance lasting 2 minutes. Ultimately, the sections were sealed using glycerin gelatin.

**Statistics analysis**

All mice were randomly divided into groups. Between the two groups of normal distribution and non-normal distribution data using student’s t-test and rank sum test, respectively. The one-way analysis of variance (ANOVA) was employed to compare the various groups. P values for the Pearson’s correlation analysis between two groups were calculated using the two-tailed test. The area of aortic lesions was quantified using ImageJ software and expressed as a percentage of the total area of the whole vessel. *P* < 0.05 was considered statistically significant. All data are presented as mean ± standard error of the mean (S.E.M.).


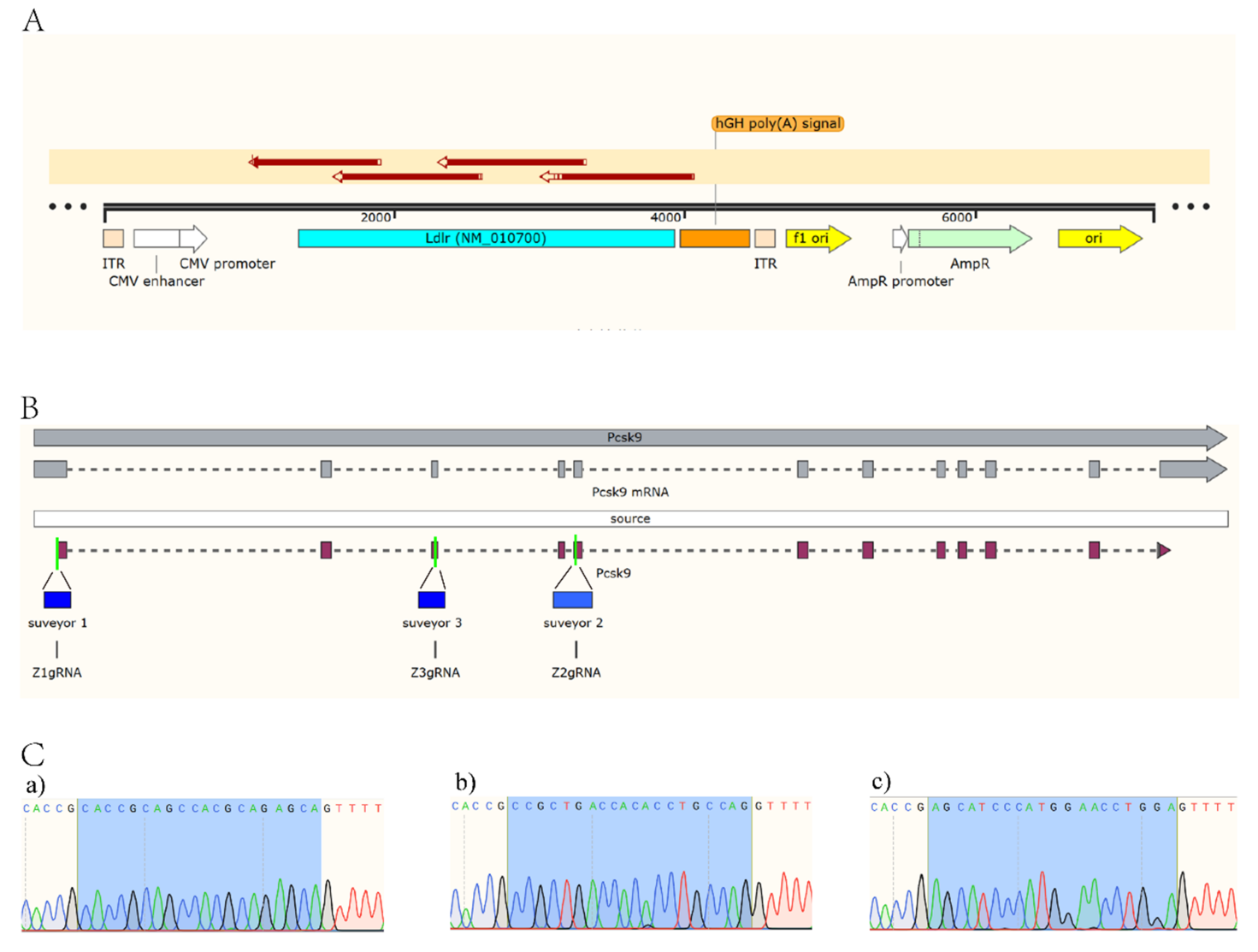


Supplementary Figure S1. Construction and validation of LDLR overexpression and PCSK9 knockout vectors. (A) Construction of the LDLR overexpression vector and validation by one-generation sequencing post-homologous recombination cloning. The mouse LDLR gene (NM_010700) was amplified by polymerase chain reaction (PCR) and cloned into a linearized AAV vector. (B) Design and construction of PCSK9 knockout vectors targeting transcripts 1, 3, and 5. The SURVEYOR assay was used for sequence verification of the target sites. (C) Sequencing validation of the guide RNA (gRNA) sequences for the PCSK9 knockout vectors, confirming the correct insertion of the gRNAs into the knockout vectors.


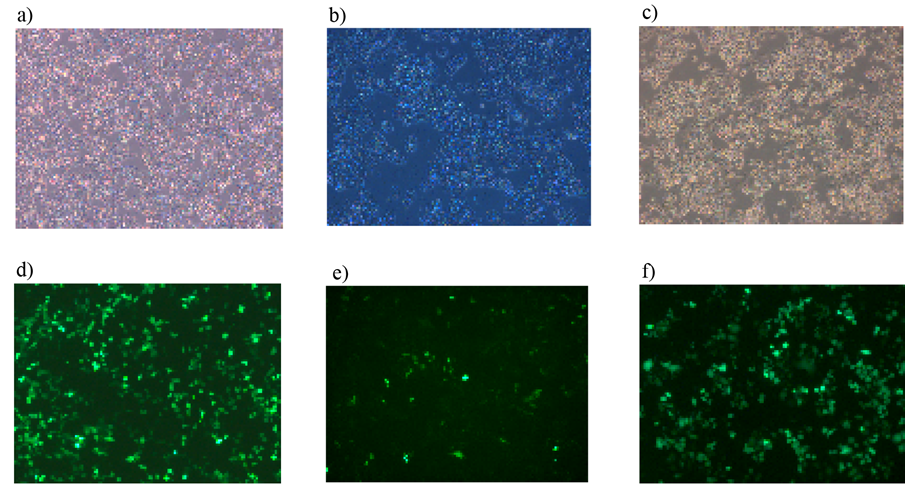


Supplementary Figure S2. Brightfield and fluorescence imaging of three cell lines (Hepa1-6, H2.35, and Neu-2a) transfected with the pAAV-EF1a-enhanced green fluorescent protein (eGFP) plasmid using nano-liposome reagent. Fluorescence imaging was performed 48 hours after transfection, confirming efficient transfection in all three cell lines. (a, d) Hepa1-6 cells; (b, e) H2.35 cells; (c, f) Neu-2a cells. The eGFP signal was used to assess transfection efficiency.


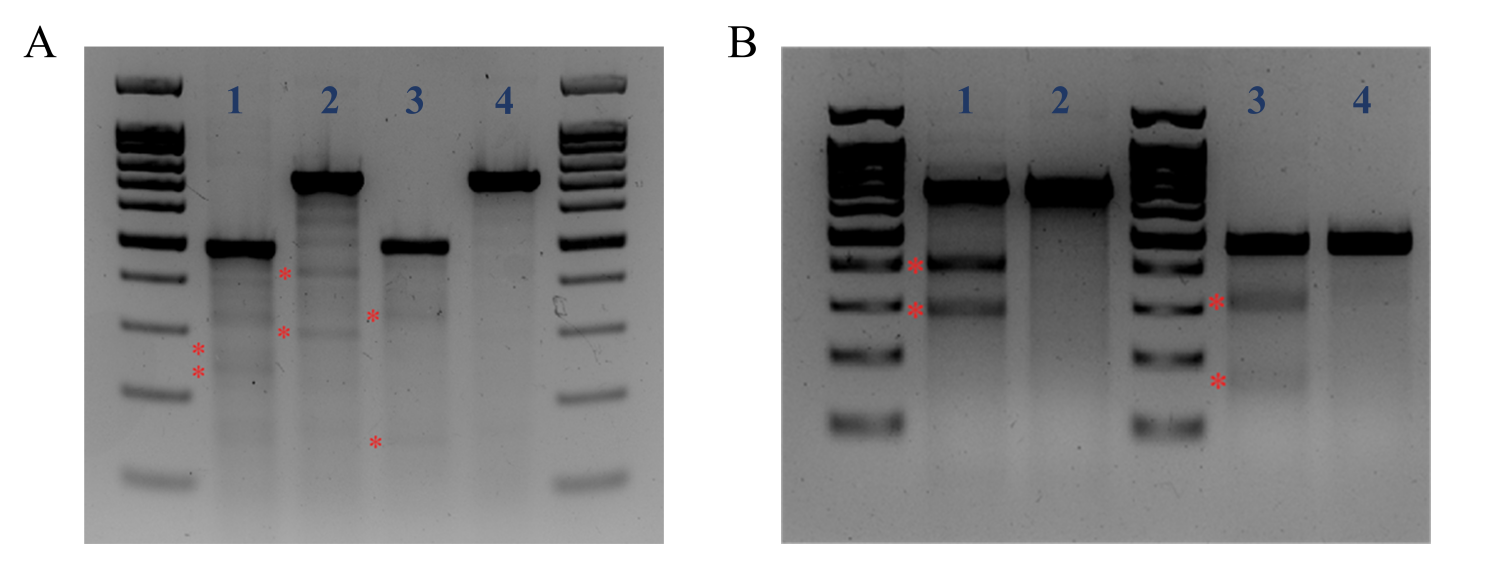


Supplementary Figure S3. Mutation detection in H2.35 and Neu-2a cells after transfection with PCSK9 knockout vectors. (A) Mutation detection by enzymatic cleavage in H2.35 cells after transfection with PCSK9 knockout vectors. The experiment utilized three designed knockout vectors (Z1, Z2, Z3) and a GFP control vector. Lane 1 represents cells transfected with vector Z1, lane 2 with vector Z2, lane 3 with vector Z3, and lane 4 with the GFP control. The enzymatic digestion patterns reveal weaker cleavage bands in lanes 1, 2, and 3 compared to lane 4, indicating successful but varying levels of gene editing. (B) Mutation detection in Neu-2a cells transfected with the PCSK9 knockout vectors Z2 (lane 1) and Z3 (lane 3). Enzymatic cleavage bands were observed in Z2 and Z3, confirming efficient gene editing, while the GFP control (lanes 2 and 4) showed no cleavage.


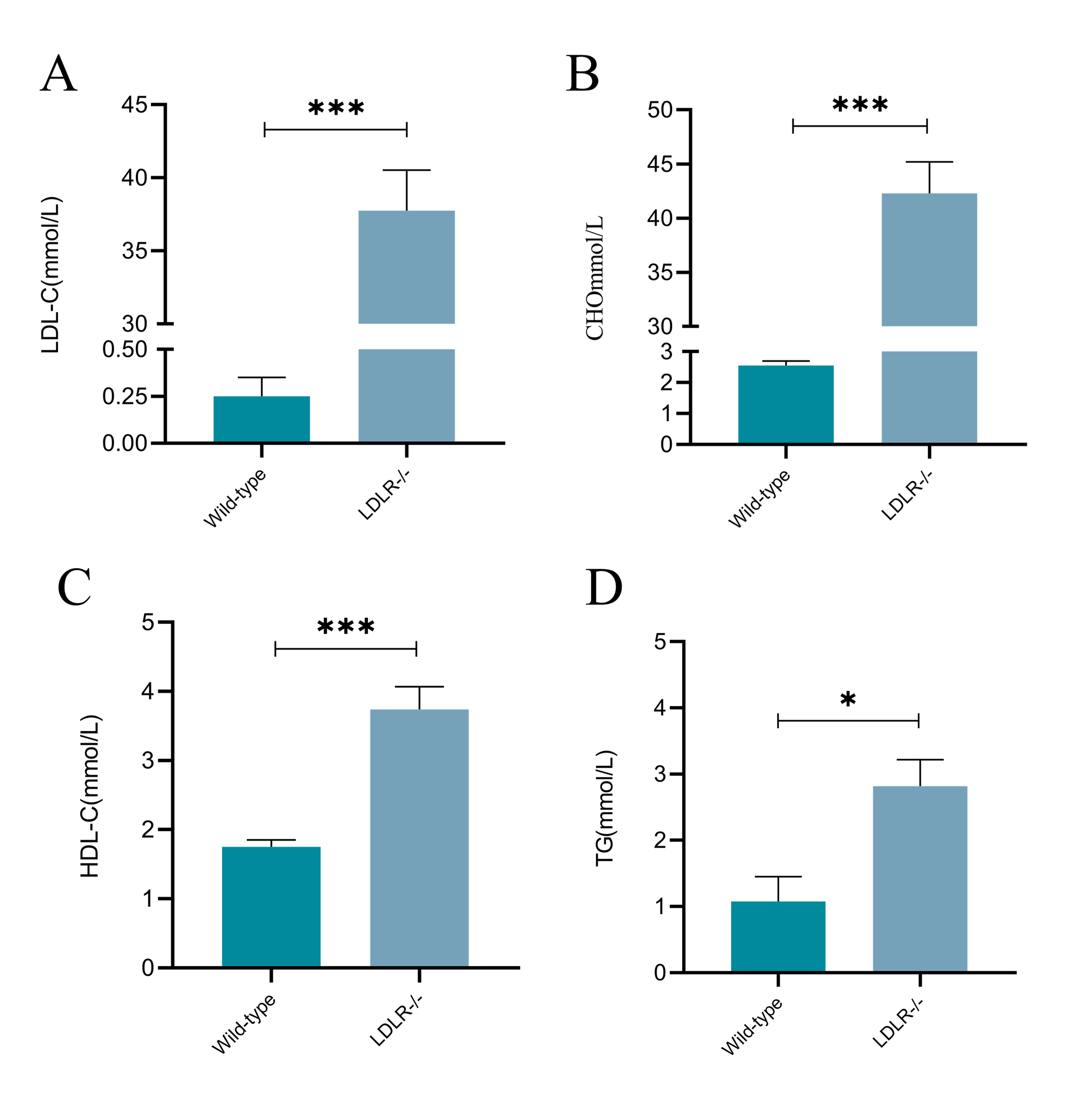


Supplementary Figure S4. Biochemical assays for atherosclerosis modeling constructs in LDLR-/- mice. (A) Serum low-density lipoprotein cholesterol (LDL-C) levels in LDLR-/- mice after 6 weeks on a high-fat diet. The data show a significant elevation in LDL-C levels, consistent with the induced atherosclerotic phenotype. (B) Serum cholesterol (CHO) levels in LDLR-/- mice on a high-fat diet. The elevated CHO levels further confirm the lipid abnormalities associated with atherosclerosis in these mice. (C) Serum high-density lipoprotein cholesterol (HDL-C) levels in LDLR-/- mice. HDL-C levels were markedly reduced in the high-fat diet group compared to controls, indicative of dyslipidemia. (D) Serum triglyceride (TG) levels in LDLR-/- mice. Elevated TG levels were also observed in the high-fat-fed mice.


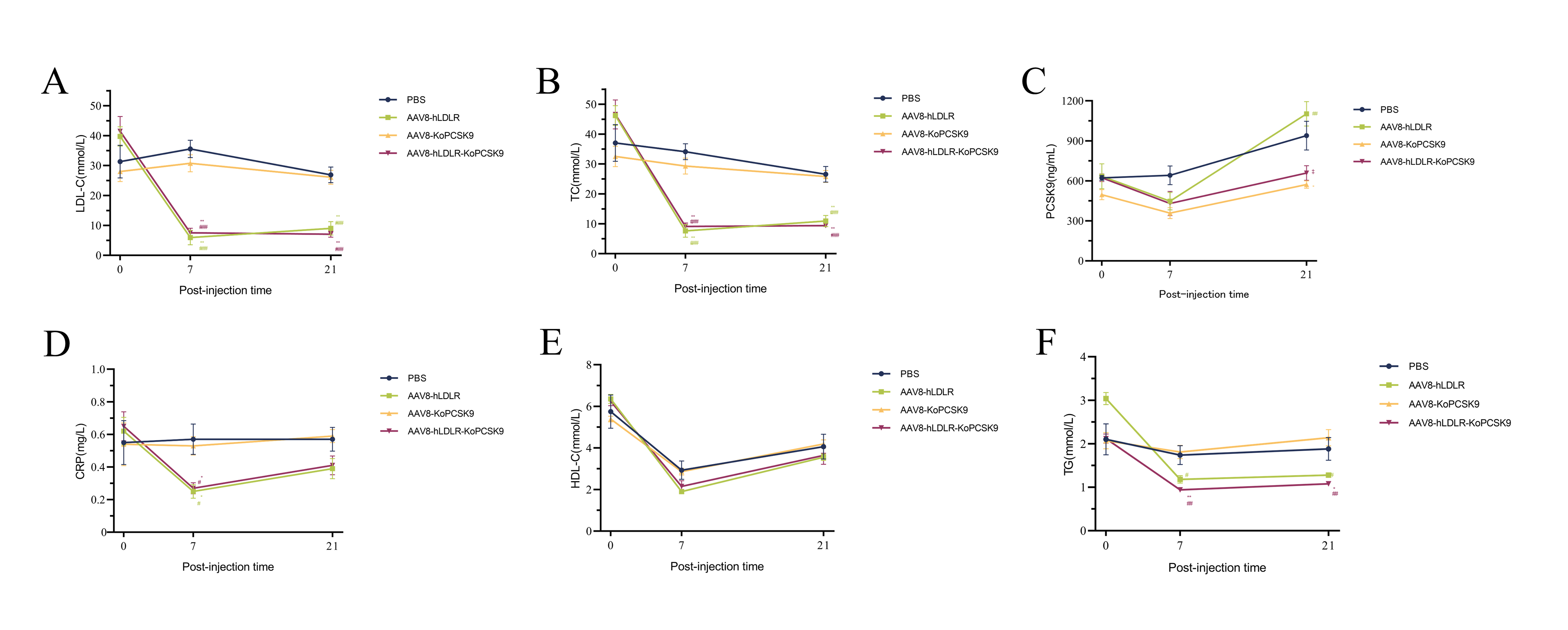


Supplementary Figure S5. Serum lipid and inflammatory marker changes in LDLR-/- mice following AAV treatment. (A) Absolute levels of LDL-C in LDLR-/- mice at 7 and 21 days following AAV treatment. Significant reductions in LDL-C levels were observed in the AAV8-hLDLR and AAV8-hLDLR-KoPCSK9 groups compared to the PBS and AAV8-KoPCSK9 groups. (B) Absolute levels of total cholesterol (CHO) in LDLR-/- mice after AAV treatment. Both the AAV8-hLDLR and AAV8-hLDLR-KoPCSK9 groups showed significant decreases in CHO levels at 7 and 21 days compared to the PBS and AAV8-KoPCSK9 groups. (C) Absolute PCSK9 levels. (D) Absolute C-reactive protein (CRP) levels. Serum CRP levels were significantly reduced at 7 days in the AAV8-hLDLR and AAV8-hLDLR-KoPCSK9 groups, with no further changes at 21 days. (E) Absolute high-density lipoprotein cholesterol (HDL-C) levels. HDL-C levels were significantly increased at 7 days in the AAV8-hLDLR and AAV8-hLDLR-KoPCSK9 groups, confirming the treatment effects on lipid profile modulation. (F) Absolute triglyceride (TG) levels. Absolute TG levels were significantly reduced in the AAV8-hLDLR and AAV8-hLDLR-KoPCSK9 groups compared to the PBS control and AAV8-KoPCSK9 groups at both 7 and 21 days, suggesting lipid-lowering effects of the treatments. The control group was the correction group. *: compared with PBS control group; #: compared with KoPCSK9 group; ‡ : compared with hLDLR group; †: 7-day versus 21-day percent change; *: *p* < 0.05; **: *p* < 0.01; ***: *p* < 0.001; error line indicates standard error of mean.


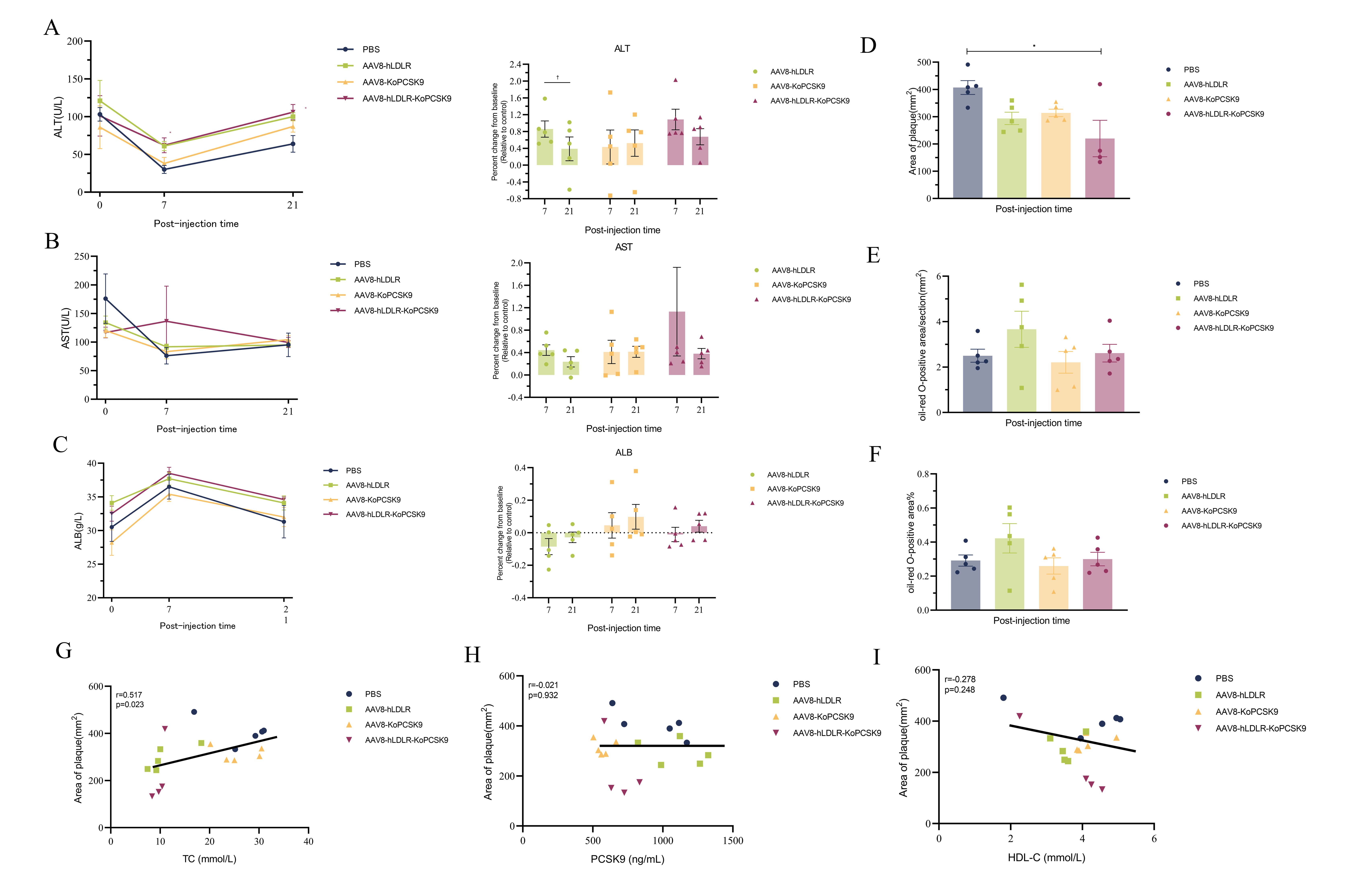


Supplementary Figure S6. Assessment of liver function, aortic plaque burden, and lipid correlations following AAV treatment in LDLR-/- mice. (A) Absolute ALT levels and percent change relative to baseline. No significant differences in ALT levels were observed between groups, except for the AAV8-hLDLR group, which showed a significant difference in the percentage change from baseline. (B) Absolute AST levels and percent change relative to baseline. No significant differences in AST levels were observed among the treatment groups. (C) Absolute albumin (ALB) levels and percent change from baseline. No significant differences were found in ALB levels between groups, suggesting that liver function was not markedly affected by the interventions. (D) Quantitative analysis of aortic root plaque area. The AAV8-hLDLR and AAV8-hLDLR-KoPCSK9 groups exhibited a significant reduction in plaque area compared to the PBS and AAV8-KoPCSK9 groups, indicating the therapeutic effects of these treatments on plaque formation. (E) Quantitative analysis of oil red O staining area. The quantitative analysis showed a significant decrease in oil red O staining area in the AAV8-hLDLR and AAV8-hLDLR-KoPCSK9 groups, consistent with the reduction in plaque lipid content. (F) Percentage of oil-red staining per unit of liver. No significant differences were observed in the percentage of oil-red staining in the liver between the treatment groups. (G) Correlation between total cholesterol (TC) levels and plaque area. A significant positive correlation was observed between serum TC levels and plaque area, indicating that higher cholesterol levels correlate with larger plaque size. (H) Correlation between PCSK9 levels and plaque area. No significant correlation was found between serum PCSK9 levels and plaque area, suggesting that PCSK9 levels may not be directly related to plaque burden in this model. (I) Correlation between HDL-C levels and plaque area. A significant negative correlation was observed between HDL-C levels and plaque area, indicating that higher HDL-C levels were associated with reduced plaque formation. *: *P* < 0.05; error line indicates standard error of the mean.
